# “Crying in the Wilderness”—The Use of Web-Based Support in Telomere Biology Disorders: Thematic Analysis

**DOI:** 10.2196/64343

**Published:** 2024-12-16

**Authors:** Emily Eidenier Pearce, Alina Majid, Toniya Brown, Rowan Forbes Shepherd, Camella Rising, Catherine Wilsnack, Ashley S Thompson, Melissa B Gilkey, Kurt M Ribisl, Allison J Lazard, Paul KJ Han, Allison Werner-Lin, Sadie P Hutson, Sharon A Savage

**Affiliations:** 1 Clinical Genetics Branch, Division of Cancer Epidemiology and Genetics National Cancer Institute National Institutes of Health Rockville, MD United States; 2 Healthcare Delivery Research Program National Cancer Institute National Institutes of Health Rockville, MD United States; 3 Trans-Divisional Research Program National Cancer Institute National Institutes of Health Rockville, MD United States; 4 Department of Health Behavior Gillings School of Global Public Health University of North Carolina at Chapel Hill Chapel Hill, NC United States; 5 Hussman School of Journalism and Media University of North Carolina at Chapel Hill Chapel Hill, NC United States; 6 Behavioral Research Program, Division of Cancer Control and Population Sciences National Cancer Institute National Institutes of Health Rockville, MD United States

**Keywords:** social media, dyskeratosis congenita, telomere biology disorder, health communication, qualitative, thematic analysis, web-based information, web-based support, telomere, biology disorder, social support, emotional support, genetic, internet-based, information-seeking, descriptive study, semistructured interview, adult, illness experience, psychosocial, digital health, health intervention, health informatics

## Abstract

**Background:**

Web-based information and social support are commonly used in rare disease communities where geographic dispersion and limited provider expertise complicate in-person support. We examined web-based resource use among caregivers of individuals with telomere biology disorders (TBDs), which are rare genetic conditions with long diagnostic odysseys and uncertain prognoses including multiorgan system cancer risk.

**Objective:**

This study explored internet-based information-seeking and social support practices and perspectives of patients with TBDs and their caregivers.

**Methods:**

Our qualitative descriptive study used semistructured interviews of patients with TBDs and caregivers. Data were transcribed verbatim and thematically analyzed by an interdisciplinary team.

**Results:**

A total of 32 adults completed interviews. Participant ages ranged from 27 to 74 years. The majority (n=28, 88%) were female, occupied multiple TBD roles (eg, patient and parent), and had undergone genetic testing. Most engaged in web-based information-seeking (n=29, 91%) and TBD-specific social media (n=26, 81%). Participants found web-based resources useful for information-seeking but reported privacy concerns and frustration with forming supportive relationships. Most participants described ambivalence toward web-based resource use, citing tensions between hunger for information versus distrust, empowerment versus overwhelm, disclosure versus privacy, and accessibility versus connection. Fluctuations in web-based support use arose from perceived harms, information saturation, or decreased relevance over the course of TBD illness experience.

**Conclusions:**

Individuals with TBDs and their caregivers reported frequent use of web-based informational and emotional support. However, ambivalence about the benefits and liabilities of web-based resources and persistent medical uncertainty may impact the adoption of and adherence to web-based support among patients with TBD and caregivers. Our findings suggest web-based psychosocial support should target long-term and multifaceted informational and emotional needs, be user-initiated, be offered alongside in-person formats, provide expert-informed information, and be attentive to personal privacy and evolving support needs of the TBD community. This study suggests web-based resources will be most effective in the TBD context when they achieve the following features: (1) offer a variety of ways to engage (eg, active and passive), (2) provide privacy protections in moderated “safe spaces” designed for personal disclosure, (3) offer separate venues for informational versus emotional support, (4) combine web-based relationship formation with opportunities for in-person gathering, (5) provide information that is reliable, easy to access, and informed by medical professionals, (6) remain mindful of user distress, and (7) are responsive to variations in levels and types of engagement. Additionally, advocacy organizations may wish to avoid traditional social media platforms when designing safe spaces for web-based emotional support, instead pivoting to internet-based tools that minimize privacy threats and limit the perpetual public availability of shared information.

## Introduction

### Background

In rare diseases, limited medical expertise and geographic dispersion of cases challenge diagnosis, access to expert care, and connection with peers [1], creating an environment where individuals with rare disease and their caregivers must become disease experts and advocates. The rapid development of web-based resources for rare diseases has made the internet a routine tool for information-seeking and community-building by clinicians, patients, and caregivers [2,3]. Emerging rare disease initiatives rely on the internet as a platform for social connection and intervention delivery, as well as to cocreate knowledge by crowdsourcing information about symptoms, access to care, treatments, and health outcomes [4,5]. However, the potential harms of web-based health misinformation, particularly in contexts of high medical uncertainty, have been repeatedly documented [6-9]. The tension between the benefits and challenges of web-based support in rare diseases raises the need to investigate patient and caregiver perspectives on the use of internet-based resources. Here, we report the perspectives of individuals with telomere biology disorders (TBDs) and their caregivers regarding TBD-related internet and social media use.

### TBDs

TBDs are rare, genetic conditions that affect both pediatric and adult populations, with most patients experiencing symptoms before the age of 20 [10-12]. Dyskeratosis congenita (DC), the classic TBD diagnosis, is identified by a triad of nail dysplasia, abnormal skin pigmentation, and oral leukoplakia; however, not all individuals with TBDs exhibit these traits. Improved understanding of TBDs has led to diagnosis in connection with a wide phenotypic spectrum and germline pathogenic variants in over 18 different genes, with features including abnormally short telomeres, bone marrow failure, pulmonary fibrosis, liver disease, high risk of certain cancers, and many other manifestations [10-13]. Individuals with TBDs often have compromised immunity as a result of either the TBD or its treatment (eg, chemotherapy, liver or lung transplant, or hematopoietic cell transplant), which can result in lengthy restrictions on social interactions [10-12]. A diagnosis of a TBD leads to a lifetime of cancer screening and monitoring for other progressive clinical manifestations across multiple organ systems [10-12]. While the genetic origin of the disease remains unknown for about 20% of affected individuals, in most known cases, TBDs are transmitted through multiple inheritance patterns. As a result, a single individual may experience multiple simultaneous roles (eg, a person may be an adult patient with a TBD and a caregiver to a child with a TBD) [14].

### Use of Web-Based Resources in Rare Disease

Rare disease advocacy organizations commonly develop web-based platforms for emotional and informational support [15-18] and advancing research, harnessing the expertise of patients and caregivers to cocreate knowledge alongside scientific collaborators [4,5,19]. Studies across a variety of health conditions support the psychosocial benefits of providing and receiving web-based support [17,20-37]. Additionally, web-based crowdsourcing is described as a positive, democratizing force that will improve the reach, relevance, and translation of scientific research [38-41]. In rare disease communities, web-based engagement has been linked to patient empowerment and improved health equity [5,19,39,42,43]. However, despite the documented benefits of web-based resources in rare diseases [32,37,44,45] and the wide-ranging application of web-based crowdsourcing for data collection [41,46], tool validation [47,48], and health communication [4,49], evaluations of the effectiveness of web-based resources to improve health outcomes have mixed results [50]. Studies suggest the impact of web-based resources may vary by type of support, user expectations, and web-based group dynamics. For example, a study of TBD-related social media posts found users engaged more often with informational posts than posts providing emotional support [51]. Another study of web-based cancer communities found participation in web-based groups decreased patient satisfaction with in-person health care; patients became aware of superior clinical experiences described by others and subsequently developed decreased satisfaction with their own clinical experiences due to social comparison [52]. A study of web-based Interactive Cancer Communication System users found patients with lower emotional well-being more often engaged with web-based resources than those with higher emotional well-being [24]. Additionally, qualitative interviews with genetic disease patients and caregivers revealed that web-based resources were considered useful for advocacy and health decision-making, but noted concerns about the privacy and trustworthiness of web-based advice [15]. Additionally, a systematic review found web-based support communities were dominated by White, adult, female participants, a pattern also noted among TBD-specific social media users [51], which limits the generalizability of existing research findings [50] and suggests restricted inclusivity of web-based spaces.

Conflicting findings on benefits and harms, combined with a lack of best practices for content, delivery, and oversight, suggest the need for additional research to define patient and caregiver expectations and experiences with web-based resources. Additionally, existing studies of web-based resource use are limited to the perspectives of individuals who are current web-based resource users, and little is known about the general expectations, acceptability, or perceived efficacy of web-based resources among individuals with rare disease and their caregivers who have refrained from engaging in web-based communities. This study aimed to examine patient and caregiver perspectives about and practices of web-based resource use in the context of TBDs, a condition whose rarity, complexity, and high degree of medical uncertainty make it especially relevant for the study of web-based support-seeking.

## Methods

### Overview

This study used semistructured, deidentified, interview transcripts from 32 individuals with TBDs and their caregivers who participated in the TBD Needs Assessment Study at the National Cancer Institute (NCI; ClinicalTrials.gov identifier: NCT04959188). The parent study was a mixed methods exploratory study of survey data and qualitative interviews conducted by the NCI in partnership with Team Telomere, Inc, an international TBD patient advocacy organization [53]. Needs assessment findings, participant characteristics, and study procedures are described in detail elsewhere [54].

### Participants and Recruitment

Eligible participants included English-speaking individuals aged 15 years or older who were diagnosed with DC or a related TBD and current or bereaved caregivers (parents, spouses, or siblings). We recruited participants between November 2021 and May 2023 from families enrolled in the Inherited Bone Marrow Failure Syndromes clinical study (NIH Protocol 02-C-0052, ClinicalTrials.gov, identifier NCT00027274) at the NCI via email, newsletters, or at clinical visits or through Team Telomere, Inc by advertisement in communications, including the monthly digital newsletter, social media (Facebook and Twitter), and at an in-person camp for families with TBDs. We also recruited participants enrolled in the NCI inherited bone marrow failure syndrome study (NIH Protocol 02-C-0052, ClinicalTrials.gov, identifier NCT00027274) via email or at clinical visits occurring at the National Institute of Health (NIH) clinical center in Bethesda, Maryland. Recruitment was not limited to individuals from the United States but extended internationally. All participant names are pseudonyms.

### Procedures

Participants engaged in semistructured telephone interviews lasting between 60 and 134 minutes between September 2021 and May 2023 conducted by one of five qualitatively trained interviewers (AST, CW, CR, RFS, and EEP) using a semistructured interview guide focused on capturing participant needs and experiences. Audio-recorded interviews were transcribed verbatim and deidentified prior to analysis.

### Data Analysis

Data analysis for this substudy followed a combined content analysis approach [55]. First, three independent coders (EEP, PKJH, and MBG) collaborated to develop a preliminary codebook (Pearce, forthcoming) that included a priori code categories for “internet use” and “social media use.” Then, 3 independent coders (EEP, AM, and TB) coded all transcripts, meeting weekly over the course of 9 months to compare coding and arrive at a consensus through discussion. Disagreements about codes were reviewed by a fifth coder (PKJH), discussed, and resolved through group consensus. One coder (EEP) then organized segments containing “internet use” or “social media use” content using software-assisted coding in MaxQDA (version 22.1.1; VERBI GmbH). Then three coders (EEP, AM, and TB) collaboratively reviewed the segments and identified themes within participant descriptions of TBD-related internet and social media use using a thematic analysis approach as described by Saldaña [56] and Miles et al [57]. Throughout the coding and analysis process, coders incorporated reflexivity prompts into their team meetings in keeping with best practices in qualitative research [58]. Themes are illustrated by anonymized participant quotations [58].

### Transparency and Openness

In compliance with the NIH data management and sharing policy, deidentified data, analysis code, and research materials are available upon reasonable request to the corresponding author after the establishment of appropriate data transfer agreements.

### Ethical Considerations

This study is a substudy of the TBD Needs Assessment study that was approved by the NIH Institutional Review Board (NIH Protocol 000502-C, ClinicalTrials.gov, Identifier NCT04959188). Written informed consent was obtained for all participants. The original Institutional Review Board allows the secondary analysis without additional consent. Study data are deidentified. Participants in the TBD Needs Assessment study were eligible for a US $25 Amazon or Target gift card if they completed both the web-based survey and telephone interview.

## Results

### Overview

A total of 32 adults participated in semistructured interviews. The majority (n=28, 88%) were female, occupied multiple TBD roles (eg, patient and parent), and had undergone genetic testing. Almost half (n=15, 47%) were either themselves or had a spouse employed in a medical field. The median time since receiving a personal or family diagnosis was 6 (IQR 0-18) years (Table 1).

**Table 1:** Participant characteristics.

		Value (N=32)
Age (years)^a^, median (IQR)		49 (27-74)
Time since TBD^b^ diagnosis (years)^c,d^, median (IQR)		6 (0-18)
**Sex, n (%)**		
	Male	4 (13)
	Female	28 (88)
Respondent or family received genetic testing for TBD, n (%)		31 (97)
Respondent or spouse ever employed in a medical field, n (%)		15 (47)
**TBD role, n (%)**		
	Multiple	23 (72)
	Bereaved	19 (59)
	Patient	17 (53)
	Parent	15 (47)
	Spouse	6 (19)
	Sibling	4 (13)

^a^n=31.

^b^TBD: telomere biology disorder.

^c^n=27.

^d^Calculated as the time between TBD diagnosis and interview date.

Nearly all participants reported using web-based resources, including TBD nonprofit webpages, Google searches, and scientific journals. Most participants also reported viewing or posting on TBD social media, with the majority using Facebook to connect with Team Telomere public or private community groups. Categories of social media engagement were assigned according to participant self-report: participants who described creating posts were considered “active,” those who described viewing but not creating posts were considered “passive,” and those who had discontinued or never initiated engagement with social media were considered “avoidant” (Table 2).

**Table 2:** Self-reported internet and social media telomere biology disorder exploration.

		Value (N=32), n (%)
Internet exploration (any)		29 (91)
**Internet exploration source (n=29)**		
	Nonprofit^a^	17 (59)
	Google	13 (45)
	Peer-reviewed journal^b^	7 (24)
	Government^c^	4 (14)
	Medical center or hospital	2 (7)
	Other^d^	7 (24)
Social media activity (any)		26 (81)
**Social media engagement type (n=26)**		
	Active	10 (41)
	Passive	9 (27)
	Avoidant	6 (38)
	Other^e^	1 (5)
**Social media activity source (n=26)**		
	Facebook^f^	17 (65)
	Instagram	2 (8)
	Linked In	2 (8)
	Other^g^	5 (20)

^a^Nonprofit sources included: Team Telomere (n=17), Be the Match (n=1), Leukemia and Lymphoma Society (n=1), and National Neutropenia Network (n=1).

^b^Peer-reviewed journal sources included: PubMed (n=2), NORD (n=1), Up-to-Date (n=1), Blood (n=1), New England Journal of Medicine (n=1), and miscellaneous (n=2).

^c^Government sources included: National Institutes of Health (n=4).

^d^Other internet exploration sources included: YouTube (n=1), Online Mendelian Inheritance in Man (n=1), own website (n=1), and undefined (n=4).

^e^Other social media engagement types included: memorialized deceased spouse’s Facebook page (n=1).

^f^Facebook groups included: Team Telomere (n=17), other dyskeratosis congenita (n=2), Myelodysplastic syndrome (n=1), Be the Match (n=2), Neutropenia (n=1), and Shwachman-Diamond (n=1).

^g^Other social media activity sources included: Caring Bridge (n=1), Marco Polo (n=1), X (formerly Twitter; n=1), the American Lung Association Inspire website (n=1), and blogs (n=1).

Overall, participants described dynamic use of web-based resources that evolved over time to meet multiple support needs (eg, informational and emotional), frequently in combination with in-person support-seeking strategies (eg, using social media to stay in contact with peers they met at in-person events, or visiting peers they initially connected with web-based). Most participants described initiating web-based resource use after diagnosis, using diagnostic terms (eg, “telomere” or “dyskeratosis congenita”) in search engines to discover informational materials and disease-specific support groups. Participants described TBD web-based communities as helping them meet others with shared illness identity and participate in activities (eg, peer gatherings) not available to them within their in-person contexts due to their geographic dispersion or immunocompromised status. However, participants expressed ambiguous feelings about web-based support tools, noting several tensions caused them to weigh competing values including (1) hunger for readily available information about TBDs versus distrust of information provided on the internet; (2) feeling empowered and comforted by web-based communities versus feeling overwhelmed by the emotional and time-related toll of web-based communities; (3) disclosing personal information to help and connect with others versus maintaining boundaries and protecting personal privacy; and (4) generalized, convenient access to the broader TBD community versus the desire to build deep, personalized peer connections specific to their individual needs. Participants explained these ambiguous feelings contributed to their disengagement from web-based resources, alongside feelings of saturation with available information and changing support needs across the TBD illness experience. We discuss these patterns in more detail below.

### Hunger for Information Versus Distrust of Web-Based Resources

#### Overview

Participants described web-based resources as central to their search for information about TBDs; however, the presence and accessibility of web-based information created tensions for many. On one hand, participants described using web-based tools to augment or clarify what they learned from in-person clinicians when they either did not fully understand or trust the information being provided. However, participants also acknowledged distrust of what they found on the internet. Overall, participants described a complex relationship with information-seeking, noting that access to multiple information sources (web-based and in-person) could increase trust when the information was consistent, but exacerbate distrust when information was contradictory.

#### “I Want The Answers Now”: Use of Web-Based Resources for Information-Seeking in TBDs

Most participants described urgent searches for information after receiving a diagnosis, which often occurred virtually via web-based patient portals. Web-based receipt of diagnosis frequently created a seamless pivot from return-of-results to web-based information searches, with participants often using diagnostic keywords in Google while waiting for their in-person follow-up medical appointments. Eliza, an individual with DC, explained that web-based searches served as a way of coping with worry while waiting for in-person appointments, “I looked up things on the internet because it’s really hard to get a hold of some doctors..” She explained:

I do tend to go to Google when I have no other resources at the time. Especially when I am worried ... I want the answers now instead of several months from now.

Even after accessing in-person care, many participants described continuing to rely on web-based resources, citing them as often more relevant, informative, and accurate than information they received from local providers. Grace, a bereaved caregiver, explained,

You need that network of people that are all virtual. Because chances are, your local clinics and hospitals are ... not going to have any idea what they’re grappling with.

Ruby, a caregiver, said:

When [the doctor] was like, “...we need to test his telomeres,” we would ask, “What’s that for?” She wouldn’t say for the diagnosis of DC. She just would say, “It’s part of the standard of testing for these gene mutations.” But we found out through Google it was to define whether he had DC or not.

#### “Everything on the Internet, Not All of it is True”: Distrust of Web-Based Information

Despite frequently accessing web-based information about TBDs, participants expressed concerns about the trustworthiness and validity of information they found on the internet. Eliza, an individual with DC, said: “Everything on the internet, not all of it is true...” Victor, a caregiver, explained:

...Going to the internet and look around for information based just on the symptoms my son had ... it was really ... nonsense ... you can find everything when you don’t have a clue.

Gloria, parent of an individual with a TBD, explained she regretted spending so much time searching the internet after her son’s diagnosis, as most of the information she found proved irrelevant:

Our minds are scanning for danger and worse possible scenarios ... doing the research and all that ... the things that we imagine, you know, in the middle of the night ... just fortunately almost never happen ... if I could’ve spared myself all of those terrifying internet searches, you know? I wish I could go back and tell myself, “Hey, you don’t need to do that.”

### Empowerment Versus Overwhelm

#### Overview

Many participants described accessing web-based information and support as an entryway into feeling more empowered and engaged in health decision-making. For many, web-based resources provided valuable platforms to connect with TBD research generated by others, as well as to engage in their own knowledge-generating activities. Many described gaining a sense of agency from information discovery, often sharing knowledge found on the internet with in-person providers to help direct their own care. However, exposure to TBD-related web-based information and support groups also led to a feeling of overwhelm. Participants described overwhelming emerging from discovering wide gaps in the scientific understanding of TBDs, the emotional impact of information, and the ever-present lure of web-based resources.

#### “Follow the Trail”: Web-Based Resources as a Source of Empowerment

Participants explained that web-based resources could be a source of empowerment, allowing them to participate more fully in their health decision-making and contribute to completing the scientific “puzzle” (Janice) of TBDs by compiling evidence or generating new knowledge. Lola, a caregiver, used web-based resources in partnership with her child’s medical care team to evaluate screening and treatment options, saying:

...Every time a new journal article would come out, I’d be like, hey. And sometimes [the doctor and I would] agree on stuff, sometimes we wouldn’t. But if I had a persuasive argument, he’d be like, “Hey, I see where you’re coming from. Let’s run this test.”

Sonia, an individual with DC, used the internet to research functional properties of proteins impacted by her genetic mutation:

...I look up TERC, and I wanted to know what this protein is ... that little, tiny fragment of a piece of the puzzle, I can do that.

Janice, a bereaved sibling, said her brother developed his own website to find others experiencing idiopathic pulmonary fibrosis, a common clinical manifestation of TBDs in adults, in collaboration with a clinical research team:

...he was in contact with a lot of patients ... if you searched for IPF, his website came up ... in a Google search ... my brother collected these questionnaires from people who contacted his website, and then he forwarded them to

Medical University researchers

#### “I Can’t do This Anymore”: Web-Based Resources as a Source of Overwhelm

Despite celebrating web-based information and community as a source of empowerment, many participants explained that exposure to web-based resources could also lead them to become overwhelmed by the quantity of content, their emotional reaction to disease information, the rapidly changing landscape of TBD science, and exhaustion from time spent on the internet.

Faith, a caregiver, said,

I read on the internet ... I study everything with TBDs ... I talk to people in support groups ... it’s very stressful, and it keeps me up at night.

She explained her ambivalence about the benefit of the web-based support group, saying:

It was really scary when I first joined the groups because ... there were parents who had just lost their children, and there was a lot of prayers going on ... I wasn’t even sure if I wanted to stay in there because I knew I needed that information and that support group, but it was also making my anxiety even worse.

Audrey, an individual with a TBD who is also a caregiver, described how web-based resources fed her mother’s anxiety and strained their relationship:

And my mom was horrible ... she reads the internet 24 [hours a day] ... I mean all night long. I mean she’d be sending me articles at 2:00 AM and I’m like, “I can’t do this.” Like I had to finally tell her, I was like, “I can’t do this anymore, you have to stop.”

### Disclosure Versus Privacy

#### Overview

Participants described feeling the tension between disclosing personal information helpful to others living with TBDs and maintaining their personal boundaries and privacy protections. Personal disclosure was a powerful source of connection for many, and several touted the benefits of web-based communities as places to anonymously “learn while lurking.” However, many said the speed and reach of web-based networks meant information-sharing could quickly breach personal boundaries, creating a sense of violated privacy and straining supportive relationships.

#### “I Like to Talk About It”: Benefits of Personal Disclosure in Web-Based Communities

Several participants identified web-based resources as an opportunity for sharing their experiences with TBDs, explaining that disclosure of their personal struggles helped them form a community with supportive others. Audrey, an individual with DC, described being a caregiver-expert in TBD web-based communities, gaining emotional support by providing information to newcomers:

I like to talk about it. I like to share these stories. I don’t know why ... it just kind of helps me release ... to help other people on their journeys and whatever they need help with.

Melissa, a caregiver, described feeling encouraged and comforted by generations of patients with TBDs and their caregivers on the internet:

I know there’s a lot of families on there ... so they’ll be there [online] ... answering questions for other parents that ... are just getting diagnosed or just have general questions.

#### “I Don't Like to be Super Open Online”: Privacy Liabilities of Web-Based Communities

Several participants noted they felt less safety in web-based networks compared to in-person relationships because it was harder to control personal boundaries. Participants noted personal privacy concerns, the stress of unpredictable emotional dynamics on web-based forums, and how the speed and fluidity of web-based communication could quickly cross boundaries of personal comfort.

Olivia, a caregiver, expressed a particular distrust of Facebook and concerns about web-based privacy in general, saying,

I’m not on Facebook ... I don’t like to be super open online ... I don’t want people to Google me and find out a bunch of other more private information.

Linda, an individual with a TBD, described feeling her privacy had been violated when her sister began sharing Linda’s health information on Facebook, ultimately causing her to decrease her own web-based presence.

She would make these posts on Facebook ... my sister came down for my transplant ... I had lost my hair, and I was starting to look very cancer patient-y. And she took these pictures. And, of course, those are the pictures she posts every year.

For Eve, an individual with DC, the fear of privacy liabilities on the internet caused her to limit her participation in support groups, preferring to be “one of the lurkers,” though she preferred to remain anonymous on the internet due to privacy concerns, she still described benefitting from web-based communities, saying,

I don’t hardly ever put anything out there. But I learn a lot from other people and what they’re experiencing.

### Generality Versus Specificity

#### Overview

Many participants described using web-based resources for generalized access to web-based TBD peer groups and crowdsourcing rare communities. While many recounted positive benefits from the ease of access to other individuals with TBDs and their families on the internet, others were ambivalent about using web-based spaces to build and inhabit a social reality alternative to their regular in-person environments. For some, web-based communities, while easily accessible, were too general and provided less specificity of connection compared to their in-person relationships.

#### “I Wasn’t Going to be Crying in the Wilderness”: Generalized Access to Web-Based Community Support

Participants described using web-based resources to build web-based networks of peers and TBD expert clinicians to achieve a sense of normality and belonging. Sarah, a bereaved spouse, described using the internet to find a connection after her husband’s diagnosis, saying,

Is there anybody else in my boat? That’s what I was trying to look for ... reaching out to somebody online...

Dina, a bereaved caregiver, explained,

...I found [Team Telomere]. I was just doing web searches ... I went to their website ... I was going to join a group of people that existed. I wasn’t going to be crying in the wilderness.

Victor, a caregiver, described Team Telomere’s web-based network as “like a family somehow.” Eve, an individual with DC, described the connection she felt on TBD-specific social media groups as comparable to a religious community:

I’m spiritual but I don’t believe in organized religion for myself ... So, I really appreciate the groups on Facebook.

Some participants described linking their web-based and in-person lives, using web-based resources to bridge emotional and practical barriers. Juno, a parent of an individual with a TBD, developed web-based group chats to continue relationships with peers she initially met in person. Faith, a caregiver, explained that she preferred relying on her web-based networks to initially process medical news before engaging with her in-person communities:

...If we get some bad news at the doctor's office ... my phone is continuously blowing up. What did you find out at the doctor? And sometimes I’m just like, okay, right now I’m just trying to take it all in ... I may get on social media and talk to some of the people in the support group about what I found out ... most of the time, it’s just staying home, not interacting with anyone else until I can figure out how to deal with it myself...

#### “What We Were Reading Didn’t Match Up With What We Were Experiencing”: Limited Specificity of Connection With Web-Based Community

Despite the helpfulness of web-based communities reported by many participants, several expressed frustrations over the shortcomings of web-based groups and noted a preference for in-person relationships. While web-based networks provided easy access to others experiencing TBDs, participants found these experiences may not match their own due to the wide-ranging TBD clinical manifestations and lack of web-based presence of specific peer groups (eg, adult patients).

Linda, an individual with DC, said,

I feel like it is so hard [to find peers online] just because it’s such ... a vast array of ... things that everyone has experienced.

Lola, a caregiver, expressed similar frustration with web-based forums, saying:

I’ll ask a question every now and then. Usually 99.9 percent of the time, nobody has anything to say about my questions, because we are so different from everybody else.

Sonia, an adult with DC, explained:

Most of the stuff I see on groups are children that have DC ... or I’m hearing from the caregiver’s perspective ... I’m not the caregiver, so it’s harder for me to relate to.

Diana, an individual with DC, explained she used web-based TBD peer relationships for information-seeking rather than psychosocial support, noting that even 13 years after diagnosis, she still felt a lack of personal connection in web-based environments:

Right now [I use online resources] more for comparing notes and searching for information. I don’t know that I’ve formed enough of a bond with anyone where I would feel comfortable talking about spirituality or some of my deep concerns or worries.

### “You Really Gotta Move On With Your Life”: Fluctuating Benefits of Web-Based Resources

#### Overview

In addition to the burden of navigating potential liabilities of web-based engagement previously discussed, participants also described changes to their web-based resource use over time as they reached saturation with existing resources or transitioned to different stages of illness.

#### Information Saturation: “I Felt Like I Knew All I Needed to Know”

Many participants who stopped using web-based resource use after periods of concentrated engagement described having reached saturation with or no longer needing web-based resources due to changes in the TBD illness experience. Audrey, an individual with a TBD, explained:

I’m like what does it matter to keep reading anymore? I don’t know that enough has changed in the last two years ... at the beginning ... I read every article I could find. I don’t do that anymore. It’s never good ... I know what I need to know at this point ... and [continuing to search online] wasn’t worth it.

#### Emotional Disengagement: “Going Quiet”

Other participants explained a need to disengage with TBD web-based resources over time after they entered new phases of life with TBDs, regardless of the availability of new information. Lola, a caregiver of an individual with DC, described discontinuing her web-based searches for information and support after her child’s illness stabilized, noting that a change in illness status would encourage her to re-engage if she needed to acquire new information:

I read what’s going on with Team Telomere. I’m not very active in it, just because ... we’re just not in that stage of our life right now ... Now, if she needed a liver transplant tomorrow, I’m sure I’d be very involved.

Others described disengaging or “going quiet” (Cynthia) on social media after bereavement. Sally, a bereaved spouse, explained:

I got involved in [Team Telomere Facebook] and that became a really good outlet for me ... I stayed involved with them for about a year [after my spouse died] ... after about a year, I said, “It’s kind of keeping you in this world and you really gotta move on with your life.”

## Discussion

### Principal Findings

This study found frequent use of web-based resources as tools for informational and emotional support-seeking among individuals with TBDs and their caregivers but also revealed ambivalence about the overall risks and benefits of web-based support that could erode web-based support use over time. Specifically, we identified several themes illustrating conflicting values and ambivalence among participants regarding the use of TBD web-based resources, including (1) hunger for information versus distrust, (2) empowerment versus overwhelm, (3) disclosure versus protection, and (4) general versus specific support needs.

Our findings on the use of web-based resources by patients with TBDs and their caregivers are in line with past research showing individuals from rare disease communities [4,5,19] use web-based resources to clarify in-person advice from medical providers, whose lack of knowledge of rare diseases may make patients skeptical of their medical recommendations [59,60]. Participant use of diagnostic and disease-specific keywords to find web-based resources supports ongoing efforts to facilitate web-based support access in rare diseases through search engine optimization [61], particularly for medically underserved populations who may rely on symptom descriptions rather than diagnostic terms in their web-based searches. Our findings on participants’ use of web-based information searches to not only gather the facts but also to relieve anxiety are consistent with past research suggesting that information-seeking may provide an overall sense of control in times of great change, irrespective of any specific desire for information [62]. Participant expressions of distrust in web-based medical information lend support to growing calls to combat misinformation a critical component of 21st-century public health [63]. This may be a particularly challenging task for patients, caregivers, and medical providers dealing with rare diseases, given that limitations in scientific knowledge of these diseases may make it difficult for people to discriminate between true and false information, regardless of their level of health- or technological-literacy [64].

The conflict between desire for information and distrust of information likely plays into the tension between viewing web-based resources as both a source of empowerment and emotional overwhelm. Participant recognition of how web-based resource use was harming them mirrored findings from adult patients with cancer [65] and parents of children with a rare genetic skin disorder, epidermolysis bullosa [66], for whom psychosocial coping improved with avoidance of, rather than engagement in, web-based forums. These findings are also in line with previously cited research linking information-seeking and anxiety, which showed that anxiety increased when information gathering provided evidence contrary to expectations and desires [62]. These findings also lend support to a recent advisory from the United States Surgeon General about the harms of social media use on youth mental health and underscore the need for caution in the development of web-based support communities, particularly for vulnerable groups [67].

Participant ambivalence about disclosing personal needs web-based is in keeping with research on TBD web-based support groups which found that most posts were unsolicited offers of support or experience-sharing and rarely included expression of negative emotions or psychosocial needs [51]. These dynamics were also found in web-based cancer groups, in which experience-sharing was more frequent than overt support requests [21]. The expressed preference of some study participants to be “lurkers” on TBD social media is in line with research in which more than half of registered users engaged passively, rather than actively, in web-based support platforms in other health contexts [36]. Concerns about personal privacy and trustworthiness of web-based information found in this study are also reflected in a growing body of research revealing inaccuracies in web-based health information and seeking to improve web-based safety in patient care [68,69]. These concerns have resulted in the emergence of recommendations that sites be monitored and moderated to ensure the accuracy of content and maintain healthy social dynamics [70]. The variable impact of web-based resource use as palliating or worsening distress has led to calls for the identification and promotion of best practices for web-based support in the cancer context [71]. Our study suggests a similar need to establish guidelines for web-based support in rare disease contexts, including acknowledgment of the complexity of web-based resource use, where users may encounter competing values that impact the frequency and extent of their engagement.

Participant assertions that web-based resource use improved their well-being align with findings from cancer care recovery [72] and other rare disease contexts, and highlight the benefit of establishing belonging in a rare disease “family” [73,74]. The importance of community belonging was also noted by Gilman [75] in a study of Fanconi Anemia, a rare, inherited cancer predisposition disorder, which showed that a disease-specific web-based group, or “imagined community,” provided alternative normality that diluted the impact of isolation, shame, and stigma encountered in in-person lived experiences. Similarly, Pearce et al [51] described how a TBD-specific Facebook community generated and perpetuated shared TBD cultural symbols (eg, unicorn motifs and “transplantiversaries”) and supported social connections that coexisted in web-based and in-person spaces. Research from other contexts examining how web-based communities may influence the formation of individual and community-level illness identities may help inform further research in rare diseases. For example, a borderline personality disorder hashtag on Instagram found that a common illness identity developed through the adoption of shared visual themes surrounding challenges to self-concept, symptoms of illness, suggested avenues for comfort, and sharing positive experiences [76]. Other research identified features of adolescent identity formation impacted by web-based communities (eg, self-presentation, social comparison, role modeling, community history, social context, and validation of self-concept) [77]; this research was informed by theories of youth identity development (Erikson [78]) and social cognitive aspects of mass communication (Bandura [79]) that could provide avenues for future exploration of rare disease identity formation web-based.

Notably, participants reported that while web-based “imagined communities” can provide a space for social acceptance and illness identity formation, their disconnection from in-person, or “in-real-life” communities may limit their supportive capacities. This may especially be the case for common social media platforms that may have few active users engaged in primarily asynchronous interactions. A study of a TBD Facebook community group found that posts were created by a minority (36%) of users and research on web-based cancer support communities found a perceived lack of peer responsiveness in asynchronous web-based platforms [23]. In this study, participants explained that while web-based resources could initially give them a sense of belonging, failure to find peer connections or to extend those connections from “web-based” to “real” life experiences could erode the supportive utility of web-based communities and exacerbate feelings of isolation. This is in keeping with research on loneliness, happiness, and depression during the COVID-19 pandemic which found positive effects of in-person social interactions (eg, household size) but no effects of web-based social interactions [80]. Future research is needed to understand whether the benefits of web-based social interactions depend on whether web-based relationships are strictly web-based or also have in-person components.

The change in TBD web-based engagement reported in this study aligns with patterns found among patients with cancer who reported high levels of engagement in web-based resources postdiagnosis that decreased over time, resulting in sporadic, unreliable web-based support networks [26]. In contexts of low medical uncertainty, discontinuation of web-based engagement may reflect the achievement of user support goals. However, the complexity and persistence of medical uncertainty documented in TBDs suggest TBD web-based resource use patterns may follow a different development path compared to other health contexts. For example, the theory of web-based social support by LeCoursiere [25] presents a four-step pathway involving (1) individual awareness raising, (2) transactional exchange of experiences with others, (3) formation of social networks, and (4) “embeddedness” or community belonging and ownership. While this process may hold for some members of the TBD web-based community, changing support needs and continued medical uncertainty may interrupt the TBD web-based support transfer process. Web-based community stability in TBDs may be particularly threatened by (1) temporary or permanent discontinued engagement following perceived emotional harm due to informational or emotional overload, (2) abandonment of web-based communities due to frustration over lack of peers with relatable illness experiences, and (3) web-based support needs that ebb and flow over the course of illness. The distrust of and inconsistent participation in web-based resources may threaten the reach and effectiveness of web-based support, suggesting a need for continued research and development of web-based interventions in the context of TBD and other rare multiorgan, complex disorders to inform recommendations about optimal engagement with web-based support.

### Implications for Practice

Our findings support the development of long-term web-based support resources with access initiated by individuals with TBDs and caregivers to meet multifaceted informational and emotional needs that change over time. Web-based information and community networks in TBDs may be viewed as part of a multimodal response to patient and caregiver needs, ideally offered in combination with in-person support, as recommended by research on social support delivery effectiveness in the cancer context [28,81]. This study suggests web-based resources will be most effective in the TBD context when they achieve the following features: (1) offer a variety of ways to engage (eg, active and passive), (2) provide privacy protections in moderated “safe spaces” designed for personal disclosure, (3) offer separate venues for informational versus emotional support, (4) combine web-based relationship formation with opportunities for in-person gathering, (5) provide information that is reliable, easy to access, and informed by medical professionals, (6) remain mindful of user distress, and (7) are responsive to variations in levels and types of engagement. Additionally, advocacy organizations may wish to avoid traditional social media platforms when designing safe spaces for web-based emotional support, instead pivoting to internet-based tools that minimize privacy threats and limit the perpetual public availability of shared information.

### Limitations

The study population was limited by the use of a self-selected convenience sample of individuals recruited using web-based tools (eg, Facebook and e-newsletters) from participants in NIH studies or members of the Team Telomere patient advocacy group. Therefore, participants in this study may be more likely to conduct internet research or participate in social media compared to the wider TBD population. Given that nearly half of the study participants were employed or had a spouse employed in the medical field, their ability to use diagnostic keywords to access relevant web-based resources may be higher compared to the general population. Additionally, the self-selecting nature of the study population could introduce bias into our sample, as those with the time and energy to participate in telephone interviews may have different characteristics compared to those who did not opt into the study. Moreover, the use of web-based resources was not the singular focus of the interview guide, and we had limited ability to devote time to follow-up questions to clarify or gather more detail about participant experiences with internet and social media use. However, the frequency with which participants discussed and expanded upon web-based resource use suggested it was a key feature of their lives with TBDs. Although this study included participants from diverse genders and races, the majority were female, White, and adults, suggesting that our results, in keeping with other social media research, are more informed by this demographic and may have limited applicability to other groups. Finally, the cross-sectional nature of this study prevents us from assessing causal relationships of observing longitudinal changes in web-based resource use; we rely on self-reported motivations and use activity, often requiring participants to remember years into the past to recount web-based resource use prior to, directly following, and in life after diagnosis.

### Conclusions

While web-based interventions continue to hold promise for TBDs, groups developing web-based support resources need to be aware of the ambivalent attitudes held by patients and their caregivers toward web-based resource use including concerns about the reliability of information, informational and emotional overwhelm, privacy threats, lack of personalized connection in web-based networks, and changing perceptions about the benefits and liabilities of web-based engagement throughout the experience of illness. Overall, patients with TBDs and their caregivers reported openness to engagement with social media and other web-based resources, but there remains considerable opportunity for optimizing these resources to improve their safety, reach, and effectiveness.

## Abbreviations

DC: dyskeratosis congenital
NCI: National Cancer Institute
NIH: National Institute of Health
TBD: telomere biology disorder

## Data Availability

The datasets generated during or analyzed during this study are available from the corresponding author upon reasonable request and review by the National Cancer Institute.
